# Retroperitoneal Endometriotic Cyst Infiltrated in the Iliopsoas Incidentally Found in a Patient with Acute Back Pain

**DOI:** 10.1155/2017/2302568

**Published:** 2017-12-19

**Authors:** Nanami Tsukasaki, Takuro Yamamoto, Akihisa Katayama, Nozomi Ogiso, Tomoharu Okubo

**Affiliations:** Department of Obstetrics and Gynecology, Japanese Red Cross Society Kyoto Daiichi Hospital, 15-749 Honmachi, Higashiyama-ku, Kyoto 605-0981, Japan

## Abstract

We describe a rare case of retroperitoneal endometriotic cyst infiltrated in the iliopsoas incidentally found in a patient with acute back pain. Endometriosis at the pelvic peritoneum, including the Douglas pouch, has been reported often; there are few reports of cystic endometriosis in the retroperitoneal cavity. Today there are various theories regarding how endometriosis occurs. By pathological findings and lesion sites of the present case, we concluded that the endometrial tissues in the menstrual blood might metastasize lymphatically and implant and form the retroperitoneal cyst.

## 1. Introduction

An endometriotic lesion occurs frequently in the ovary, pelvic peritoneum, and Douglas pouch but rarely at other sites. In recent years, an endometriotic lesion that occurs at an atypical site has been defined as scarcity endometriosis in Japan. We describe a case of a retroperitoneal endometriotic cyst that infiltrated the iliopsoas and was incidentally found in a patient with acute back pain. Here, we describe the patient's clinical course and pathological findings and discuss the pathogenic mechanisms.

## 2. Case Presentation

**Figure 1:**
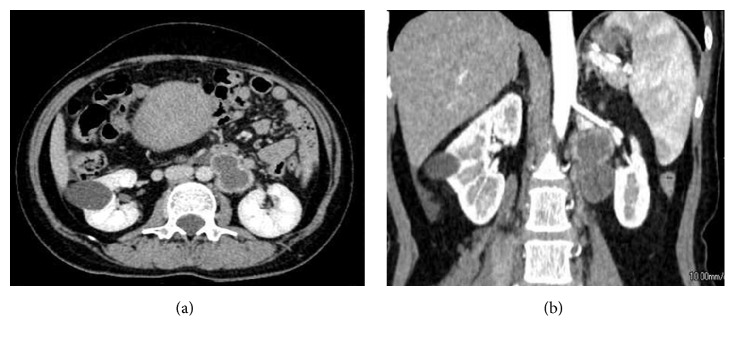
Computed tomography scan showing that the bilocular cystic mass is located caudally from the left renal hilus without solid parts.

**Figure 2:**
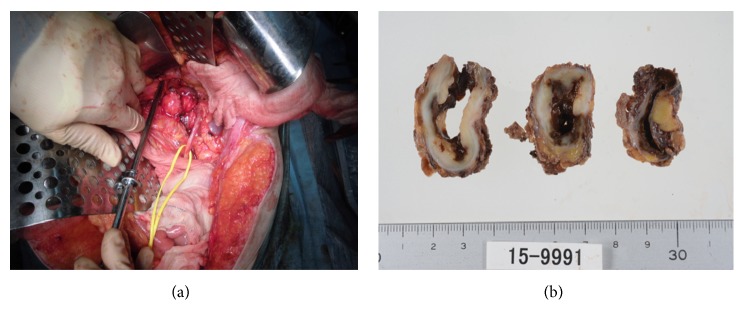
(a) Intraoperative findings showing that the peritoneal cyst in the left inferior kidney is bilocular, and it is firmly adhered to the left iliopsoas and ureter. (b) Photographs of the resected retroperitoneal cyst showing that it is bilocular with cystic space with old and new bleeding.

The patient was a 43-year-old, gravida 0, para 0, woman with a history of leiomyomectomy at the age of 28 years. She felt sudden pain between the left lower abdomen and lower back 3 days before her first visit, and she took analgesic drugs; however, the pain persisted. Originally, she did not have additional menstrual disorders and the lumbar backache was not related to menstruation cycle. The computed tomography (CT) scan showed enlargement of the uterus, which led us to suspect uterine sarcoma and para-aortic lymph node metastasis that infiltrated the iliopsoas. She was referred to our hospital for a detailed examination. Her abdomen was enlarged 5 cm above the umbilicus, and we recognized the left costovertebral angle knock pain. The transvaginal ultrasonogram showed a great mass with echo-free space in the uterus, and we suspected uterine fibroma. The serum levels of CA-125 and CA-19-9 were increased to 514.3 U/mL and 299.2 U/mL, respectively. However, the levels of CEA and lactate dehydrogenase were within normal ranges. The pelvic magnetic resonance imaging scan showed adenomyosis of the uterus and a mass lesion measuring 19 × 15 × 7 cm on the left side of the uterus with many cystic and hemorrhagic cavities. The mass had low-intensity enhancement on T1-weighted and T2-weighted images, and it did not have decreased diffusion. The bilocular cystic mass with hemorrhaging was found in the retroperitoneal cavity. The retroperitoneal cyst was 5.3 × 2.7 × 4.0 cm and did not have solid parts, and it had high-intensity enhancement on T1-weighted and T2-weighted images with shading. Therefore, we diagnosed the patient as having endometriotic cysts. Using contrast-enhanced CT, we found that this bilocular cystic mass was located caudally from the left renal hilus. The cranial cystic wall was relatively thick; however, it did not have solid parts (Figures 1(a) and 1(b)). The cyst was close to the left renal artery cranially, left ureter caudally, left kidney laterally, and left psoas major muscle medially. Therefore, we suspected that the cyst had adhered to peripheral tissues and infiltrated the left psoas major muscle, which resulted in transformation. There was no metastasis to the lung, bone, or lymph nodes (mediastinal, axillary, subclavian, and so on). All these findings led us to suspect degenerated uterine fibroma and hemorrhagic cyst growth in the retroperitoneum; thus, we performed total abdominal hysterectomy and bilateral salpingo-oophorectomy and retroperitoneal cystectomy. The surface of the uterus was smooth, and there were no malignant findings macroscopically. Both adnexa had no abnormal findings. The result of cytology of ascites was negative, and the intraoperative rapid pathological diagnosis was leiomyoma. The peritoneal cyst in the left inferior kidney was bilocular, and it adhered firmly to the left iliopsoas and ureter (Figure 2(a)). We exfoliated the cyst from the peripheral tissues being careful not to disrupt the stream of the left renal artery and ureter, and we removed the cyst by resecting a part of the iliopsoas that was infiltrated. During this process, a part of the cyst perforated, and chocolate-like fluid leaked out. There were no disseminated lesions or lymph node swelling. Therefore, we concluded that the lesions were uterine fibroma and an endometriotic cyst had formed in the left inferior renal hilus. Her postoperative course was uneventful, and she was discharged on postoperative day 7. The serum levels of CA-125 and CA19-9 were normalized to 5.7 U/mL and 8.2 U/mL and lumbar backache was improved. Pathologically, the final diagnosis was leiomyomas and endometriotic cysts in the retroperitoneal space. So far, we have not seen recurrent findings.

## 3. Pathologic Findings

### 3.1. Gross Findings

**Figure 3:**
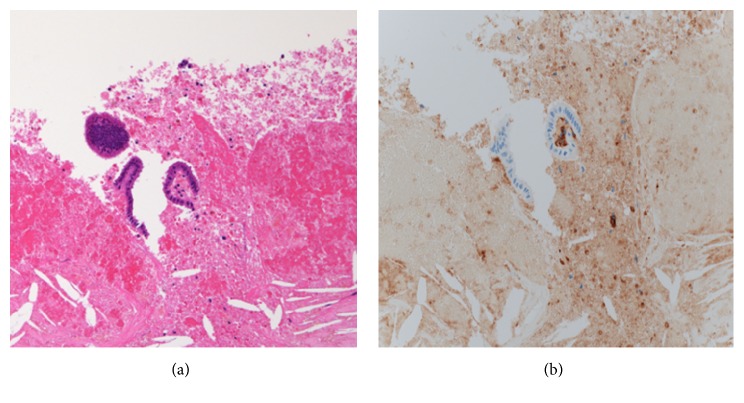
Microscopic findings using hematoxylin and eosin (HE) staining (a) and CD10 (b). We identified the endometrial gland tissues using HE staining and CD10-positive stromal cells using immunohistochemical staining.

Gross findings showed a 22-cm mass at the uterine corpus. Poorly marginated adenomyosis existed in the left side of the uterus (Figure 2(b)). The retroperitoneal cyst was bilocular, and it had cystic space with old and new bleeding (Figure 3(a)).

### 3.2. Light Microscopy Findings

The mass of the uterine corpus was associated with bleeding, and hyalinizing and eosinophilic spindle cells grew with a fascicle-like structure. Adenomyotic tissues existed in the surrounding area. The retroperitoneal cyst had exfoliated epithelia, and most cystic walls were fibrous tissues with bleeding and hemosiderosis. However, we recognized the endometrium-like tissues and endometrial stromata with CD10 expression (Figure 3(b)).

## 4. Discussion

Atypical endometriosis that occurs at sites other than female genitals has been defined as scarcity endometriosis, which develops at various sites, such as the intestine, bladder, urinary duct, diaphragm, chest cavity, and abdominal wall. Various symptoms occur depending on the original site and invasion depth. Intestinal endometriosis occurs most frequently among those with scarcity endometriosis, and the common sites are the rectum and sigmoid colon [1]. Although endometriosis at the pelvic peritoneum, including the Douglas pouch, has been reported often, there are few reports of cystic endometriosis in the retroperitoneal cavity. Since endometrial stromal cells express CD10 during proliferation, secretion, and the atrophic stage of endometrium and endometriotic lesions, CD10 is a useful marker for diagnosing endometriosis, including ectopic endometriosis [2–4]. This case pathologically showed that the epithelium was exfoliated remarkably, and most of the cystic wall was covered with fibrous tissues with bleeding and hemosiderosis. We made a diagnosis of cyst endometriosis based on the identification of endometrial gland tissues with hematoxylin and eosin staining and CD10-positive stromal cells.

There are various theories regarding how endometriosis occurs, such as the implantation theory, coelomic metaplasia theory, embryonic residual theory, hematogenous dissemination theory, and lymphatic dissemination theory [5, 6]. These hypotheses are not compatible, and it is thought that some of the mechanisms contribute to the generation of endometriosis. The coelomic metaplasia theory states that mesothelial cells and stromata on the peritoneum and ovarian surface metamorphose into endometrium-like tissues where the endometriotic tissues occur. The embryonic residual theory states that endometriotic tissues occur from the residual tissue of Müllerian and Wolffian ducts. The backflow of menstrual blood occurs in approximately 90% of reproductive females; therefore, the implantation theory makes sense in that menstrual blood moves back into the fallopian tubes and reaches the peritoneal cavity, and the endometrial tissues in the menstrual blood become implanted in the peritoneum [7]. However, in our patient, endometriosis occurred in the retroperitoneal cavity, which contradicts the implantation theory. Corpus uterine cancer frequently metastasizes lymphatically. Our patient's metastasis occurred at a very rare site of the left inferior renal hilus, which corresponds with the 325 type b lymph nodes that were the metastatic site of uterine corpus cancer. There were some lymph nodes around the retroperitoneal cystic mass pathologically. All these findings led us to conclude that the endometrial tissues in the menstrual blood metastasized lymphatically in the left inferior renal hilus and implanted and formed the retroperitoneal cyst. Similar to the present case, when physicians recognize hemorrhagic cysts in the retroperitoneal cavity in patients with lumbar backache, it is necessary to suspect an endometriotic cyst in the retroperitoneal cavity as a differential diagnosis even if there are no other findings of endometriosis. To definitively diagnose endometriosis, it is necessary to perform a pathological examination with immunostaining and to not overlook few endometrial epithelia and stromata.
